# Time to follow‐up of an abnormal mammogram in women with diabetes: a population‐based study

**DOI:** 10.1002/cam4.892

**Published:** 2016-10-06

**Authors:** Syed Yaser Habeeb, Kinwah Fung, Hadas D. Fischer, Peter C. Austin, Lawrence Paszat, Lorraine L. Lipscombe

**Affiliations:** ^1^ Department of MedicineUniversity of TorontoTorontoCanada; ^2^ Institute for Clinical Evaluative SciencesTorontoCanada; ^3^ Institute of Health PolicyManagement and EvaluationUniversity of TorontoTorontoCanada; ^4^ Sunnybrook Health Sciences CentreTorontoCanada; ^5^ Women's College Research InstituteWomen's College HospitalTorontoCanada

**Keywords:** Breast Cancer, cancer screening, diabetes, follow‐up, mammogram

## Abstract

Women with diabetes have a higher breast cancer incidence and mortality. They are also significantly less likely to undergo screening mammography and present with more advanced stage than women without diabetes. The purpose of this study was to examine if women with diabetes are more likely to have delays in follow‐up of abnormal mammograms, compared to women without diabetes. Using population‐based health databases, this retrospective cohort study examined women between the ages of 50 and 74, with and without diabetes, living in the province of Ontario, Canada, who underwent screening through a centralized program and who had an abnormal mammogram between 2003 and 2012. We compared rates of follow‐up of a diagnostic test within 180 days, as well as likelihood of mastectomy or excision procedure and a diagnosis of breast cancer. Following an abnormal screening mammogram, 97.5% of women with diabetes had a diagnostic procedure within 180 days compared to 97.9% of women without diabetes. After adjustment for other factors, women with diabetes were only 3% less likely to have follow‐up testing after an abnormal mammogram than women without diabetes (hazard ratio [HR] 0.97, 95% CI: 0.96–0.99, *P* < 0.001). The majority of Ontario women who underwent screening mammography through a centralized screening program had timely follow‐up of an abnormal mammogram, with no meaningful delays in those who had diabetes. The results of this study suggest that diagnostic delays after screening do not significantly contribute to higher breast cancer mortality in women with diabetes.

## Introduction

Evidence suggests that women with diabetes are more likely to be diagnosed with postmenopausal breast cancer, present at an advanced stage, and have increased all‐cause mortality after being diagnosed with breast cancer, compared to women without diabetes 1, 2, 3, 4, 5, 6. Although effective screening has been shown to decrease breast cancer mortality in postmenopausal women, those with diabetes are less likely to undergo regular mammograms 7, 8, 9, 10, 11. Even if a screening mammogram is performed, an abnormal test needs to be followed‐up in a timely manner in order for the screening to be considered effective.

Delays in follow‐up and diagnosis are associated with an increase in rate of cancer progression and decrease in survival 12, 13, 14. Previous studies have identified various factors as barriers to timely follow‐up of abnormal mammograms, including poor self‐rated health, limited health care access, rural residence, age, race, socioeconomic status, lack of coordinated care, and competing health care demands 15, 16, 17, 18, 19, 20, 21, 22, 23, 24, 25, 26. In addition, there is accumulating literature suggesting that the competing demands of complex, chronic diseases such as diabetes interfere with attention to other unrelated clinical services 27, 28, 29, 30. While several studies have shown lower rates of mammograms in women with diabetes, no study to our knowledge has examined whether diabetes is a barrier for adequate follow‐up of abnormal mammograms 7, 8, 11, 31, 32.

The aim of this study was therefore to examine if women with diabetes are more likely to have delays in follow‐up of abnormal mammograms, compared to women without diabetes. In addition, we examined whether other factors such as burden of chronic disease, socioeconomic status, immigration status, and rural residency influence the association between diabetes and follow‐up of abnormal mammograms.

## Subjects and Methods

### Study design and population

This was a retrospective, population‐based, matched‐cohort study, of women between the ages of 50 and 74, with and without diabetes, living in the province of Ontario, Canada. We included those who participated in the Ontario Breast Cancer Screening Program (OBSP) and had an abnormal screening mammogram result between 1 January 2003 and 30 June 2012. The age group was chosen to reflect Cancer Care Ontario's guidelines for breast cancer screening, which advise a bilateral mammogram every 1 to 3 years 33. The OBSP offers time‐appropriate breast cancer screening for all asymptomatic eligible Ontario women; approximately 60% of screening is performed through this service 34. Physician referral is not required to participate in the program and all participants are automatically notified of screening results. Women who had a previous mastectomy, a diagnosis of breast cancer, or resided in a long‐term care facility were excluded from the study.

### Data sources

We used population‐based health care databases from the Institute for Clinical Evaluative Sciences (ICES), which provide data on all residents in Ontario covered by the Ontario Health Insurance Plan (OHIP). The OHIP database includes information on physician visits and services. The Registered Persons Database (RPDB) provides demographic and vital statistics on all residents of Ontario. The Canadian Institute for Health Information (CIHI) Discharge Abstract Database (DAD) records diagnostic and procedure information from Ontario hospitalizations. The Immigration, Refugees and Citizenship Canada's Permanent Resident Database (IRCC‐PR) identifies recent immigrants to Canada.

Women with an abnormal mammogram were identified using the Ontario Breast Screening Program (OBSP) database as described above. An abnormal mammogram was defined as a recommendation for further testing by the screening radiologist 35. Patients with a previous diagnosis of breast cancer were identified from the Ontario Cancer Registry (OCR). Patients with a record in the Ontario Diabetes Database (ODD) were classified as having diabetes, based on a validated algorithm using hospitalization or physician claims records 36. These datasets were linked using unique encoded identifiers and analyzed at the ICES.

### Procedures

All women in Ontario aged 50–74 years who had an abnormal mammogram recorded in the OBSP between 1 January 2003 and 30 June 2012 were identified. The date of each subject's first abnormal mammogram was the index date for entry into the study. Next, women who had diabetes based on inclusion in the Ontario Diabetes Database prior to index were selected. Each woman with diabetes was then matched on age to two women without diabetes who had an abnormal mammogram during the same year.

### Outcomes

The primary outcome was an appropriate follow‐up diagnostic test after an abnormal mammogram, which may include a second mammogram, ultrasound, MRI, fine‐needle aspiration, or core biopsy 37. Our primary outcome was defined as the first record of one of these tests in the OHIP claims and OBSP databases within 180 days after the date of the abnormal mammogram. We chose 180 days as the maximum follow‐up duration because over 97% of women in Ontario are diagnosed with breast cancer within 6 months following an abnormal mammogram result 37. We also examined the cumulative incidence of follow‐up diagnostic testing at the following time windows: 14, 35, 49, 60, and 90 days. Secondary outcomes were a mastectomy or excision procedure within 180 days, and a diagnosis of breast cancer in the Ontario Cancer Registry within 180 days after an abnormal screening mammogram.

### Other covariates

Covariates included recent immigration, rural residence, socioeconomic status, and comorbidities. Subjects were identified as recent immigrants if they had resided for less than 10 years in Canada. They were identified as having a rural residence based on postal codes. Socioeconomic status was based on median neighborhood income levels. We categorized individuals into neighborhood income quintiles by linking their postal code data with Canadian census data on median household income levels by neighborhood of residence 38, 39. Weighted comorbidity scores were derived using the Johns Hopkins Adjusted Clinical Group Case‐Mix assignment software and a two‐year look‐back period 40, 41. Data on specific comorbidities were obtained from the OHIP database and the CIHI‐DAD, to identify patients with ischemic heart disease, congestive heart failure, hypertension, chronic kidney disease, chronic obstructive pulmonary disease, and stroke, using previously validated algorithms 42, 43, 44, 45, 46, 47.

### Statistical analyses

We used descriptive statistics to compare baseline characteristics between women with and without diabetes. We used Cumulative Incidence Functions (CIFs) to estimate the incidence of follow‐up testing, after accounting for the competing risk of death, in women with and without diabetes separately 48. For our primary analyses, we used a cause‐specific hazard model to examine whether women with and without diabetes have different rates of follow‐up testing after adjusting for patient characteristics. The robust sandwich‐type, variance estimator was used to account for the matched nature of the cohort. We performed a similar analysis for secondary outcomes to compare the hazard of follow‐up diagnosis of breast cancer or mastectomy procedure between women with and without diabetes. Multivariable regression models were adjusted for socioeconomic class, rural residence, weighted comorbidity score, and recent immigration to Canada.

### Ethics

The Institutional Review Board at Sunnybrook Health Sciences Centre in Toronto approved this study.

## Results

A total of 225,907 women met the inclusion criteria. For this study we included the 28,171 women who had diabetes at the time of the abnormal mammogram; they were then matched 1:2–56,342 women without diabetes (total 84,513). Baseline characteristics for both groups of women are presented in Table 1. The mean age at inclusion was 61 years for both groups. Women with diabetes were more likely to have immigrated to Canada in the last 10 years, belong to a lower socioeconomic status, and have a higher weighted comorbidity score than those without diabetes.

**Table 1:** Baseline characteristics of women with diabetes and age‐matched controls without diabetes, who had an abnormal mammogram

	Diabetes *n* = 28,171	No diabetes *n* = 56,342
Demographics		
Mean age at index (years)	61.19 ± 6.82	61.18 ± 6.82
Socioeconomic status quintile^a^		
Socioeconomic status 1 (lowest)	6126 (21.7%)	8691 (15.4%)
Socioeconomic status 2	6040 (21.4%)	10,537 (18.7%)
Socioeconomic status 3	5747 (20.4%)	11,163 (19.8%)
Socioeconomic status 4	5340 (19.0%)	12,104 (21.5%)
Socioeconomic status 5 (highest)	4783 (17.0%)	13,682 (24.3%)
Recent immigrant (<10 years)^b^	1206 (4.3%)	1798 (3.2%)
Rural residence^c^	3796 (13.5%)	8486 (15.1%)
Comorbidities		
Hypertension	19,194 (68.1%)	24,059 (42.7%)
Congestive heart failure	1262 (4.5%)	710 (1.3%)
Ischemic heart disease	3624 (12.9%)	3176 (5.6%)
Chronic obstructive pulmonary disease	1546 (5.5%)	1780 (3.2%)
Chronic kidney disease	1358 (4.8%)	697 (1.2%)
Stroke or TIA	786 (2.8%)	732 (1.3%)
Other previous cancer	1585 (5.6%)	2576 (4.6%)
Mean duration of diabetes (years)	6.87 ± 5.24	N/A
Mean ADG comorbidity score^d^	12.27 ± 10.95	8.21 ± 9.73

a Based on neighborhood median household income derived from census data and postal codes.

b Rural residence defined as a dissemination area with less than 10,000 residents.

c Recent immigrant defined as immigration to Ontario less than 10 years prior to index date.

d Based on physician claims and hospitalizations in the previous 2 years from Johns Hopkins Aggregated Diagnosis Groups (ADG).

Following an abnormal screening mammogram, 97.5% of women with diabetes had an appropriate diagnostic procedure within 180 days compared to 97.9% of women without diabetes (Table 2). A diagnostic test was performed within 14 days of the mammogram in 50.8% of women with diabetes and 51.4% of women without diabetes (Table 3). The median time interval between the abnormal mammogram and a follow‐up diagnostic test was 14 days (IQR 8–22) in both groups (Table 4). The first test was a mammogram, ultrasound, or both in 97.2% of women with diabetes and 97.6% of women without diabetes. An excision or mastectomy was performed in 9.6% of women with diabetes and 8.8% without diabetes, and breast cancer was diagnosed in 6.3% and 5.9% with and without diabetes, respectively. Of those women diagnosed with breast cancer, the median time interval from abnormal mammogram to diagnosis in women with diabetes was 32 days (IQR 20–52) compared to 30 days (IQR 17–49) in women without diabetes (Table 4).

**Table 2:** Proportion of women with and without diabetes who had a follow‐up diagnostic test or procedure within 180 days

	Diabetes *n* = 28,171	No diabetes *n* = 56,342
At least one follow‐up procedure or diagnostic test within 180 days	27,472 (97.5%)	55,137 (97.9%)
Additional mammogram and ultrasound (on same date)	13,862 (49.2%)	27,590 (49.0%)
Additional mammogram	9479 (33.6%)	18,643 (33.1%)
Ultrasound	4045 (14.4%)	8756 (15.5%)
Excision or mastectomy within 180 days	2697 (9.6%)	4940 (8.8%)
No follow‐up within 180 days	699 (2.5%)	1205 (2.1%)

**Table 3:** Cumulative incidences in percentages of all follow‐up diagnostic tests in women with and without diabetes (confidence intervals in parentheses)

Days from index date to follow‐up	Diabetes (%)	No Diabetes (%)	*P*‐Value
14	50.8 (50.19–51.35)	51.4 (50.95–51.77)	0.1
35	88.7 (88.30–89.05)	89.6 (89.31–89.81)	<0.001
49	93.8 (93.52–94.08)	94.5 (94.31–94.69)	<0.001
60	95.4 (95.11–95.60)	96.0 (95.85–96.18)	<0.001
90	96.8 (96.57–96.99)	97.3 (97.14–97.41)	<0.001
180	97.5 (97.36–97.72)	97.9 (97.75–97.99)	<0.001

**Table 4:** Duration to follow‐up in women with diabetes and women without diabetes

	Diabetes		No diabetes		*P*‐value	
	Median (IQR)	Mean ± SD	Median (IQR)	Mean ± SD	Median	Mean
Days from index date to follow‐up	14 (8–22)	18.24 ± 15.84	14 (8–22)	17.87 ± 15.07	0.07	0.001
Days from index date to breast cancer diagnosis	32 (20–52)	41.31 ± 31.99	30 (17–49)	38.43 ± 30.98	<0.001	0.002

Using cause‐specific hazard models, women with diabetes had slightly lower rates of subsequent diagnostic testing after abnormal mammograms than women without diabetes (Table 5). The adjusted hazard ratio (HR) of follow‐up for women with compared to without diabetes was 0.97 (95% CI: 0.96–0.99, *P* < 0.0001). There was a statistically significant difference in rates of breast cancer diagnosis between the two groups with women with diabetes being more likely to be diagnosed with cancer (HR 1.07, 95% CI: 1.01–1.14, *P* = 0.02). Women with diabetes were also modestly more likely to have an excision or mastectomy than those without diabetes (HR 1.09, 95% CI: 1.04–1.14, *P* = 0.0006). Note the univariate HR was identical to the multivariable HR, and therefore the findings are unlikely due to overfitting. There were also modest statistical differences in follow‐up based on rural residence, income, immigration status, or comorbidity (Table 5).

**Table 5:** Adjusted cause‐specific regression analysis for the association between specified parameters and first follow‐up diagnostic test

	Hazard ratio^a^	95% CI	*P*‐value
Presence of diabetes	0.97	0.96–0.99	<0.0001
Low SES	0.97	0.96–0.99	0.0001
Urban Residency	1.14	1.12–1.16	<0.0001
ADG weighted score	1.00	1.00–1.00	0.03
Recent immigrant	0.87	0.84–0.91	<0.0001

a Adjusted for age, socioeconomic status, comorbidity, urban residency, and immigration status.

## Discussion

Women with diabetes are more likely to present with advanced‐stage breast cancer and have a higher mortality after breast cancer compared to women without diabetes 5, 6. This large, population‐based study examined whether delays in follow‐up of abnormal mammograms in women with diabetes might contribute to this excess mortality. In a universal health care setting among women undergoing screening mammography through a centralized program, we found that over 95% of women had appropriate follow‐up of an abnormal test within 6 months and 50% of tests done within 14 days. Although women with diabetes were 3% less likely to have follow‐up testing after an abnormal mammogram compared to women without diabetes, we did not find any clinically meaningful delays in follow‐up for women with diabetes even when we adjusted for age, socioeconomic status, rural residence, burden of comorbidities, and immigration status.

For those women who were diagnosed with breast cancer, the majority were diagnosed within 1 month and having diabetes was associated with only a median 2‐day delay in diagnosis. Our findings suggest that, in a screened population enrolled in a centralized breast cancer screening program, diabetes is not a significant barrier to follow‐up of abnormal mammograms.

This is the first study to our knowledge that assessed the influence of chronic disease on follow‐up of abnormal mammograms. Most studies have explored the influence of sociodemographic and health care factors. Poor self‐rated health, limited health care access, rural residence, age, race, socioeconomic status, lack of coordinated care, and competing health care demands have all been shown to decrease follow‐up rates of abnormal mammograms 15, 16, 17, 18, 19, 20, 21, 22, 23, 24, 25, 26. In contrast, our study found only modest effects of sociodemographic variables, rural residence, or comorbidity on follow‐up of abnormal mammograms. This finding may be because our study was conducted within a universal health care access setting, which may have minimized these barriers. It is possible that the presence of a chronic disease such as diabetes might contribute to diagnostic delays in other health care settings, and further studies are warranted.

We found high overall follow‐up rates after an abnormal mammogram, which are consistent with other recent reports. A 2011 American study found that the median time from abnormal screening mammogram to follow‐up was 13 days, which is comparable to our result of 14 days 25. In that report, 1% of abnormal screening mammograms did not receive any follow‐up within 180 days, compared to 2.3% of women in the present study. These high overall follow‐up rates may have minimized any differences due to diabetes or other factors (ceiling effect). Our population was also limited to women who sought breast screening through the Ontario Breast Cancer Screening Program. This program has a dedicated approach to recall and follow‐up of patients, and women who use this service may have higher overall health literacy and are more likely to advocate for follow‐up. This may explain both the high rates of follow‐up and lack of differences between risk groups.

Previous studies have found that diabetes poses a significant barrier to adequate breast cancer screening 7, 8, 11, 31, 32. Our group has shown that women with diabetes are less likely to undergo screening mammography than those without diabetes, even within the Ontario Breast Cancer Screening Program 11. However, this study did not show a similar trend for diagnostic testing once women were screened. This may be because the highest risk for care gaps related to competing health care demands is during the referral process and access to appropriate services, rather than follow‐up after services have been accessed. Our population was also limited to women who had already received breast cancer screening, who may be those at lower risk of inadequate care. Those patients and providers who are compliant with routine screening may also be more apt to ensure timely follow‐up of abnormal results.

The strengths of this study include the use of validated algorithms and databases, and a large, well‐characterized, and multiethnic population receiving health care through a uniform delivery system. However, there are limitations. First, we were not able to assess the influence of important factors such as diabetes control, breast cancer risk factors, or provider practice patterns. As a corollary, we cannot determine whether rates of false positives or missed cancers differ in patients with diabetes. Second, because of the nature of a retrospective cohort study, our findings cannot exclude selection bias – women who volunteer to undergo screening mammography are also more likely to follow‐up with abnormal results. Furthermore, by limiting the primary outcome to the first diagnostic test after an abnormal screen, the study does not address the issue of incomplete follow‐up or quality of follow‐up. In addition, because the study was conducted using population‐based health care databases, data on BMI and education were unavailable. Finally, our population was limited to women who had their mammograms performed through the Ontario Breast Cancer Program in whom close to 100% follow‐up was assured. We were not able to determine whether similar trends exist for those women who had their mammogram through traditional radiology centers.

In summary, our study found that the vast majority of Ontario women who received a mammogram through a centralized breast screening program have timely follow‐up of an abnormal mammogram with no meaningful differences based on diabetes. These findings are reassuring, and do not support diagnostic delays after screening as a significant contributor to higher breast cancer mortality in women with diabetes. As our study was limited to a dedicated breast cancer screening program with universal health care access, further studies will be needed to examine this question in other health care settings.

## Conflict of Interest

The authors declare that they have no conflict of interest.
